# Supplementary material for: Reference-free assembly of long-read transcriptome sequencing data with RNA-Bloom2
Source: Nat Commun. 2023 May 22;14:2940. doi: 10.1038/s41467-023-38553-y (PMC10202958; doi:10.1038/s41467-023-38553-y)
Supplement: Supplementary file 1 — Supplementary Information [file 41467_2023_38553_MOESM1_ESM.pdf]

Reference-free assembly of long-read transcriptome sequencing data with RNA-Bloom2

Ka Ming Nip[1,2], Saber Hafezqorani[1,2], Kristina K. Gagalova[1,2], Readman Chiu[1], Chen Yang[1,2], René L. Warren[1], Inanc Birol[1,3]

[1] Canada's Michael Smith Genome Sciences Centre, BC Cancer, Vancouver, BC, Canada V5Z 4S6
[2] Bioinformatics Graduate Program, University of British Columbia, Vancouver, BC, Canada V5Z 4S6
[3] Department of Medical Genetics, University of British Columbia, Vancouver, BC, Canada V6T 1Z3

SUPPLEMENTARY INFORMATION

Supplementary Tables 3

Supplementary Table 1. Data for evaluating error correction and digital normalization. 3
Supplementary Table 2. Error rates of input reads and RNA-Bloom2 corrected reads. 3
Supplementary Table 3. Effect of digital normalization on reads aligned against assembly. 4
Supplementary Table 4. Gene representation in raw, corrected, and digitally normalized reads. 4
Supplementary Table 5. Features of the simulated data. 5
Supplementary Table 6. Peak memory usage on simulated data. 5
Supplementary Table 7. Total wall-clock runtime on simulated data. 5
Supplementary Table 8. Features of spike-in control data. 6
Supplementary Table 9. BUSCO completeness for the Sitka spruce transcriptome. 6
Supplementary Table 10. Alignment statistics of the Sitka spruce transcriptome assembly. 6
Supplementary Table 11. Number of contigs assembled for simulated data. 7
Supplementary Table 12. Number of contigs assembled for spike-in control data. 7
Supplementary Table 13. Expression of genes discarded and retained after error correction and digital normalization. 8

Supplementary Figures 9

Supplementary Figure 1. Comparing RNA-Bloom2 and isONcorrect on simulated data. 9
Supplementary Figure 2. Expression-stratified assembly recall on simulated cDNA data. 10
Supplementary Figure 3. Expression-stratified assembly recall on simulated dRNA data. 11
Supplementary Figure 4. K-mer multiplicity threshold for error correction. 12
Supplementary Figure 5. Read depth tracking with strobemers. 13
Supplementary Figure 6. Trimming and splitting based on read depth. 14
Supplementary Figure 7. Overlap graph pruning based on poly(A) information. 15
Supplementary Figure 8. Isoform assembly precision on simulated data. 16
Supplementary Figure 9. Transcripts detected per gene in simulated data. 17
Supplementary Figure 10. Transcripts detected per gene in spike-in control data. 18
Supplementary Figure 11. SQANTI3 results for three ONT cDNA mouse replicates. 19
Supplementary Figure 12. SQANTI3 results for three ONT dRNA mouse replicates. 20

Supplementary Figure 13. SQANTI3 results for three PacBio CCS mouse replicates. 21
Supplementary Figure 14. Splice junction proportions in RNA-Bloom2 assemblies. 22
Supplementary Methods 23
Supplementary Method 1. Adapter trimming for LRGASP data. 23
Supplementary Method 2. Measuring error rates. 23
Supplementary Method 3. Measuring alignment rates. 23
Supplementary Method 4. Simulation of cDNA and dRNA reads. 24
Supplementary Method 5. Extraction of spike-in control reads. 25
Supplementary Method 6. Assembly of simulated data, spike-in data, and replicated data. 26
Supplementary Method 7. Sitka spruce transcriptome analysis. 28
Supplementary Method 8. Edge filtering in the overlap graph. 31
Supplementary Method 9. Determining gene representation in sequencing reads. 32
Supplementary Method 10. Running RNA-Bloom2 and isONcorrect. 33
Supplementary Method 11. Running SQANTI3 v5.1 34

Supplementary Tables

Platform	File accession	Reads	Percentage aligned (%)
ONT dRNA	ENCFF349BIN	2,153,439	95.58
ONT cDNA	ENCFF232YSU	13,127,667	78.66
PacBio CCS	ENCFF313VYZ	2,144,172	95.49
Illumina	ENCFF696TCH<br>ENCFF751FTE	2 × 40,225,298	N/A

Supplementary Table 1. Data for evaluating error correction and digital normalization. Replicates for four sequencing platforms of a mouse dataset from the LRGASP Consortium are used. Adapters in the ONT cDNA sample were trimmed with Pychopper (See Supplementary Method 1); only full-length and rescued reads are kept. Adapter-trimming was not performed for the ONT dRNA and PacBio CCS samples because no adapters were detected. Long reads were aligned to the reference genome using minimap2 (See Supplementary Method 3). Adapters in Illumina paired-end reads were trimmed with Trimmomatic; only paired output reads after trimming are retained (See Supplementary Method 1).

Platform	Correction	Error rate (%)			
		Total	Mismatch	Insertion	Deletion
ONT dRNA	None	12.17	3.64	3.08	5.44
	Long only	10.28	3.01	2.46	4.80
	Hybrid	6.55	1.98	1.55	3.02
ONT cDNA	None	7.18	2.51	1.55	3.12
	Long only	4.03	1.43	0.85	1.74
	Hybrid	3.51	1.29	0.76	1.46
PacBio CCS	None	1.96	0.53	0.66	0.77
	Long only	1.35	0.49	0.49	0.37
	Hybrid	1.34	0.49	0.48	0.36

Supplementary Table 2. Error rates of input reads and RNA-Bloom2 corrected reads. Error correction of long reads was done in RNA-Bloom2 using either only long reads and a hybrid of long and short reads. Error rates are measured by Trans-NanoSim (See Supplementary Method 2).

Platform	Correction	Reads remaining after digital normalization (%)	Raw reads aligned against assembly (%)
ONT dRNA	Long only	48.15	97.44
	Hybrid	38.80	97.54
ONT cDNA	Long only	3.76	73.52
	Hybrid	3.53	74.02
PacBio CCS	Long only	11.66	95.16
	Hybrid	11.63	95.04

Supplementary Table 3. Effect of digital normalization on reads aligned against assembly.
Assemblies were performed with and without hybrid correction using Illumina reads. The percentage of long reads remaining after digital normalization is equal to the number of long reads remaining after digital normalization divided by the total number of long input reads to the assembly. Long input reads are aligned to each assembly with minimap2 to calculate the percentage of reads aligned (See Supplementary Method 3).

Platform	Number of genes represented				Percentage of genes retained
	Raw reads	Corrected reads		Digitally normalized reads	
ONT dRNA	20,158	Long only	19,737	19,280	95.6
		Hybrid	19,840	19,400	96.2
ONT cDNA	17,050	Long only	16,596	16,503	96.8
		Hybrid	16,891	16,758	98.3
PacBio CCS	17,853	Long only	17,748	17,365	97.3
		Hybrid	17,732	17,363	97.3

Supplementary Table 4. Gene representation in raw, corrected, and digitally normalized reads.
Gene expression is measured with Trans-NanoSim (See Supplementary Method 9) in raw long reads, error-corrected long reads, and digitally normalized long reads. The number of genes with non-zero expression is counted for each set of reads.

Feature		ONT dRNA	ONT cDNA
N50 read length (nt)		1,548	916
Error rate (%)	Total	11.88	7.42
	Mismatch	3.58	2.56
	Insertion	2.98	1.60
	Deletion	5.31	3.26
Transcripts	18 million-read set	32,664	39,313
	10 million-reads set	32,454	36,910
	2 million-reads set	28,643	27,278

Supplementary Table 5. Features of the simulated data.
Error rates are measured by Trans-NanoSim (See Supplementary Method 2).

Type	Read count (million)	RNA-Bloom2	RATTLE	StringTie2	StringTie2_GTF	FLAIR
cDNA	2	**21.7**	27.01	27.64	27.45	**60.62**
	10	42.52	135.04	**28.53**	28.71	**284.15**
	18	65.63	243.05	28.71	**28.70**	**507.12**
dRNA	2	43.61	71.2	31.26	**30.47**	**72.77**
	10	142.97	210.05	33.70	**30.39**	**333.45**
	18	154.47	377.27	34.00	**30.47**	**592.36**

Supplementary Table 6. Peak memory usage on simulated data.
Memory usage values are measured in GB; the best and worst values are highlighted in blue and red, respectively. StringTie2_GTF denotes execution of StringTie2 assisted by transcriptome annotation.

Type	Read count (million)	RNA-Bloom2	RATTLE	StringTie2	StringTie2_GTF	FLAIR
cDNA	2	0.25	**7.05**	**0.12**	0.15	0.26
	10	1.38	**31.25**	**0.43**	0.44	0.91
	18	2.90	**40.26**	0.93	**0.87**	1.30
dRNA	2	0.88	**8.09**	0.17	**0.14**	0.28
	10	7.15	**75.28**	0.70	**0.55**	1.36
	18	15.98	**147.31**	1.23	**1.00**	1.83

Supplementary Table 7. Total wall-clock runtime on simulated data.
Runtime values are measured in hours; the best and worst values are highlighted in blue and red, respectively. StringTie2_GTF denotes execution of StringTie2 assisted by transcriptome annotation.

Feature		ONT dRNA	ONT cDNA	PacBio CCS
Reads		26,814	404,783	151,982
N50 read length (nt)		1,181	712	2,460
Error rate (%)	Total	11.10	6.34	2.03
	Mismatch	3.27	2.22	0.30
	Insertion	2.77	1.45	0.81
	Deletion	5.06	2.67	0.91

Supplementary Table 8. Features of spike-in control data.
Error rates are measured by Trans-NanoSim (See Supplementary Method 2).

BUSCO category	Reads	RNA-Bloom2 (ONT only)	RNA-Bloom2 (ONT + Illumina)	RATTLE
Complete (%)	73.4	76.4	**87.6**	68.3
Complete and single-copy (%)	32.0	**38.4**	32.0	59.8
Complete and duplicated (%)	41.4	38.0	**55.6**	8.5
Fragmented (%)	7.7	7.4	**3.4**	10.2
Missing (%)	18.9	16.2	**9.0**	21.5

Supplementary Table 9. BUSCO completeness for the Sitka spruce transcriptome.
Adapter-trimmed reads, RNA-Bloom2 assemblies, and RATTLE assembly were evaluated based on 1,614 BUSCO groups in Embryophyta odb10 (See Supplementary Method 7). The best values for each BUSCO category are highlighted in blue.

Transcripts	Count
Assembled	68,514
Aligned	66,866
Split-aligned	21,423
Split-aligned with at least 1 read pair support	13,376
Unaligned	1,648

Supplementary Table 10. Alignment statistics of the Sitka spruce transcriptome assembly.
Sitka spruce ONT cDNA data was assembled with RNA-Bloom2 (See Supplementary Method 7). Illumina reads were provided for error correction of ONT reads within RNA-Bloom2. The assembly was aligned to the draft genome assembly on NCBI with minimap2. Read pair support of split alignment was verified based on STAR alignments of short reads.

Type	Read count (million)	RNA-Bloom2	RATTLE	StringTie2	StringTie2_GTF	FLAIR
cDNA	2	15,023	16,398	10,495	16,968	**21,410**
	10	25,316	34,554	19,036	27,849	**40,267**
	18	29,390	44,906	22,669	32,128	**49,484**
dRNA	2	19,854	20,030	15,442	22,322	**23,330**
	10	40,174	**44,851**	24,420	30,480	41,259
	18	48,919	**60,724**	27,716	32,322	49,190

Supplementary Table 11. Number of contigs assembled for simulated data.
Highest values are highlighted in bold.

Type	RNA-Bloom2	RATTLE	StringTie2	StringTie2_GTF	FLAIR
ONT cDNA	**993**	303	141	141	320
ONT dRNA	**314**	98	86	108	124
PacBio CCS	**249**	141	110	139	138

Supplementary Table 12. Number of contigs assembled for spike-in control data.
Highest values are highlighted in bold.

Protocol	Method	Genes	N	Expression levels (TPM)						
				min	lower whisker	Q1	median	Q3	upper whisker	max
ONT dRNA	hybrid	discarded	585	0.0	0.5	0.5	0.5	0.5	0.5	4.9
		retained	16,465	0.0	0.0	2.0	10.2	36.0	87.1	15,560.7
	long reads only	discarded	705	0.0	0.5	0.5	0.5	0.5	0.5	3.9
		retained	16,345	0.0	0.0	2.0	10.7	36.5	88.3	15,560.7
ONT cDNA	hybrid	discarded	996	0.0	0.0	0.1	0.1	0.2	0.4	46.5
		retained	19,162	0.0	0.0	0.4	2.5	12.0	29.5	14,062.3
	long reads only	discarded	1,081	0.0	0.0	0.1	0.1	0.2	0.4	46.5
		retained	19,077	0.0	0.0	0.4	2.5	12.1	29.7	14,062.3
PacBio CCS	hybrid	discarded	609	0.0	0.0	0.5	0.5	1.5	2.9	51.2
		retained	17,244	0.0	0.0	2.0	13.7	53.9	131.9	4,025.5
	long reads only	discarded	593	0.0	0.0	0.5	0.5	1.5	2.9	51.2
		retained	17,260	0.0	0.0	2.0	13.7	53.9	131.9	4,025.5

Supplementary Table 13. Expression of genes discarded and retained after error correction and digital normalization.

Gene expression was quantified by Trans-NanoSim using raw reads from ONT cDNA, ONT dRNA, and PacBio CCS. Error correction was performed using either long reads only or a hybrid of long and short reads. The number (N) of genes retained or discarded after error correction and digital normalization are reported. The minimum (min), lower whisker, first quartile (Q1), median, third quartile (Q3), upper whisker, and maximum (max) expression levels in transcripts per million (TPM) for the corresponding genes are reported.

Supplementary Figures

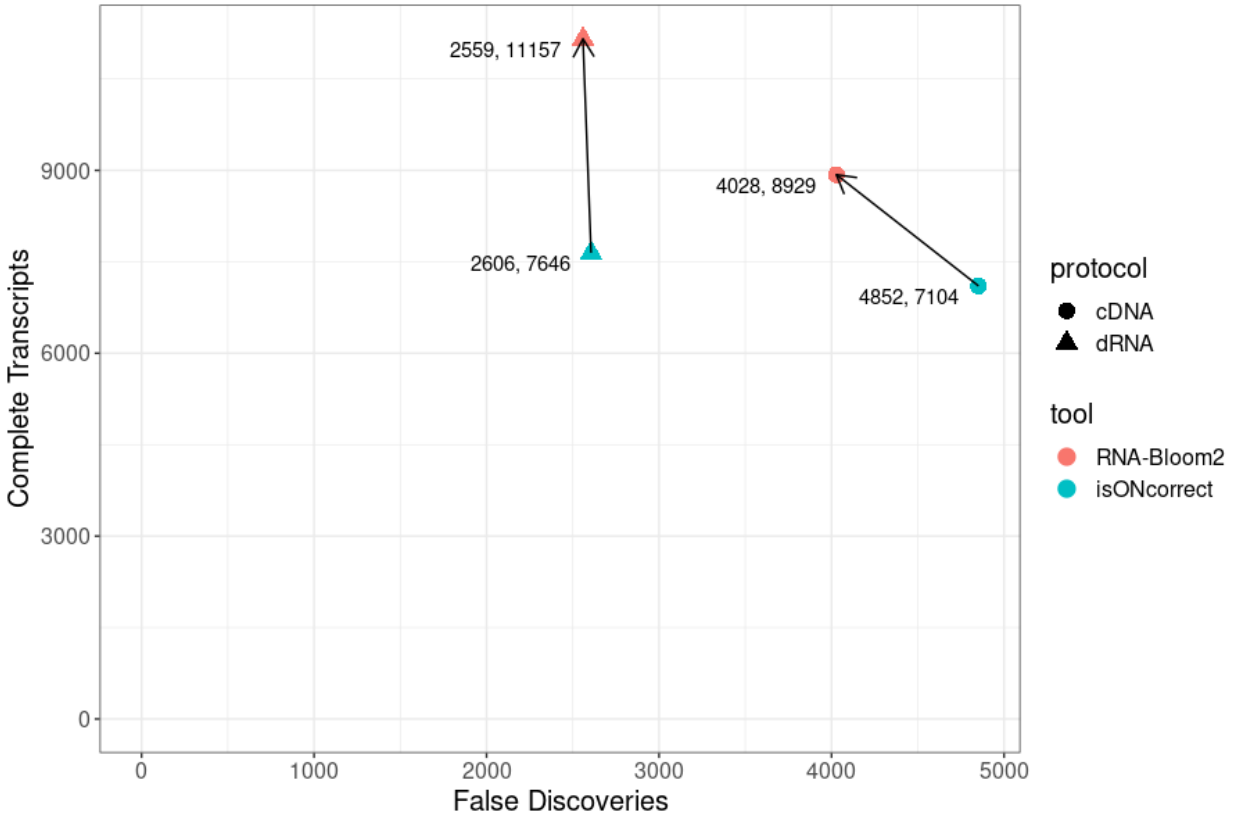

Supplementary Figure 1. Comparing RNA-Bloom2 and isONcorrect on simulated data. The number of complete transcripts and false discoveries detected in RNA-Bloom2 assemblies and isONcorrect error-corrected reads of two-million simulated cDNA and dRNA read data. The features of these simulated data are described in Supplementary Table 5. The arrows indicate the magnitude of improvement offered by RNA-Bloom2 compared to isONcorrect. Source data are provided as a Source Data file.

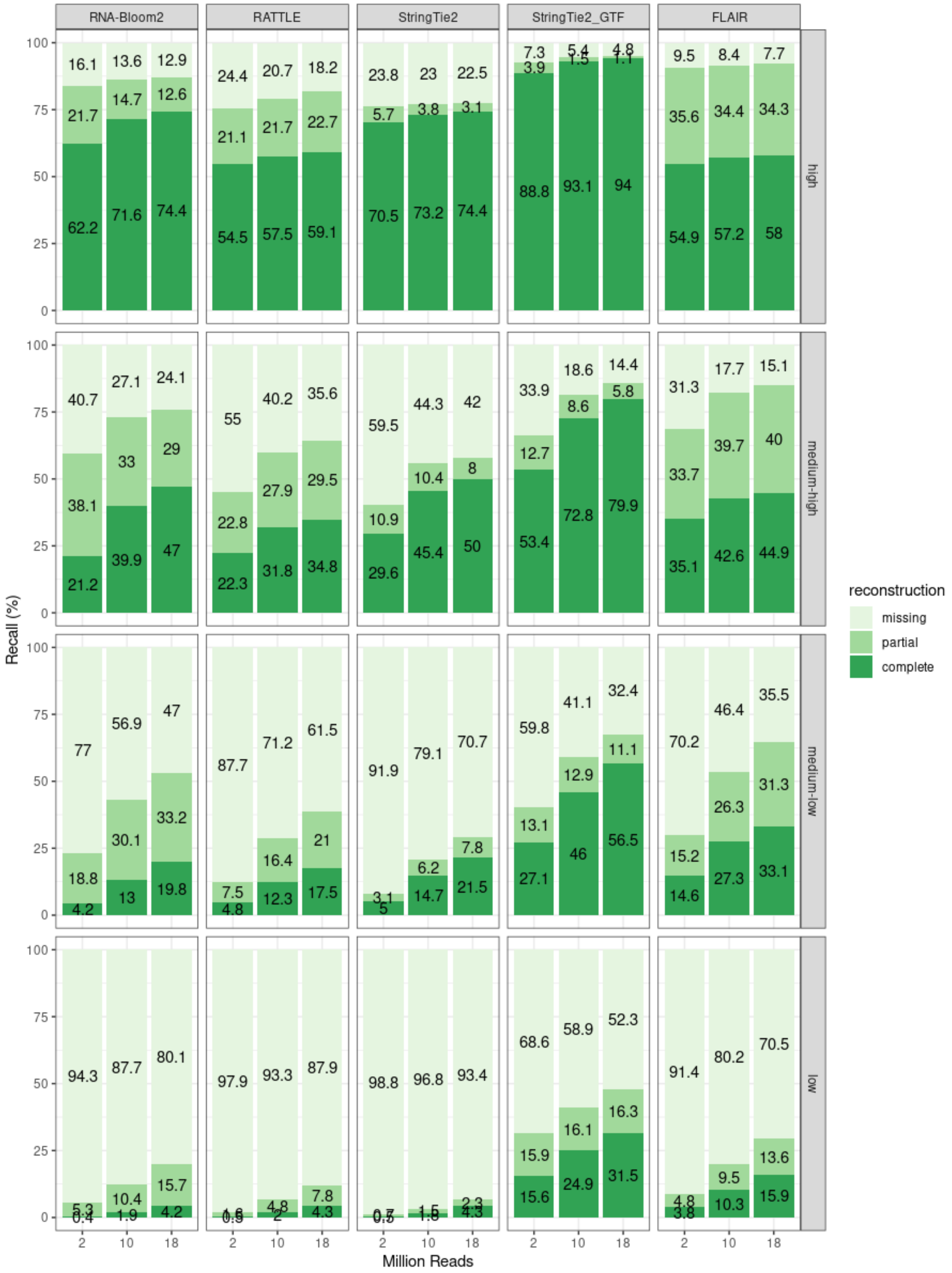

Supplementary Figure 2. Expression-stratified assembly recall on simulated cDNA data. Recall is categorized based on transcript reconstruction levels: missing, partial, and complete. Transcript expression levels are split into quartiles: low, medium-low, medium-high, and high. Source data are provided as a Source Data file.

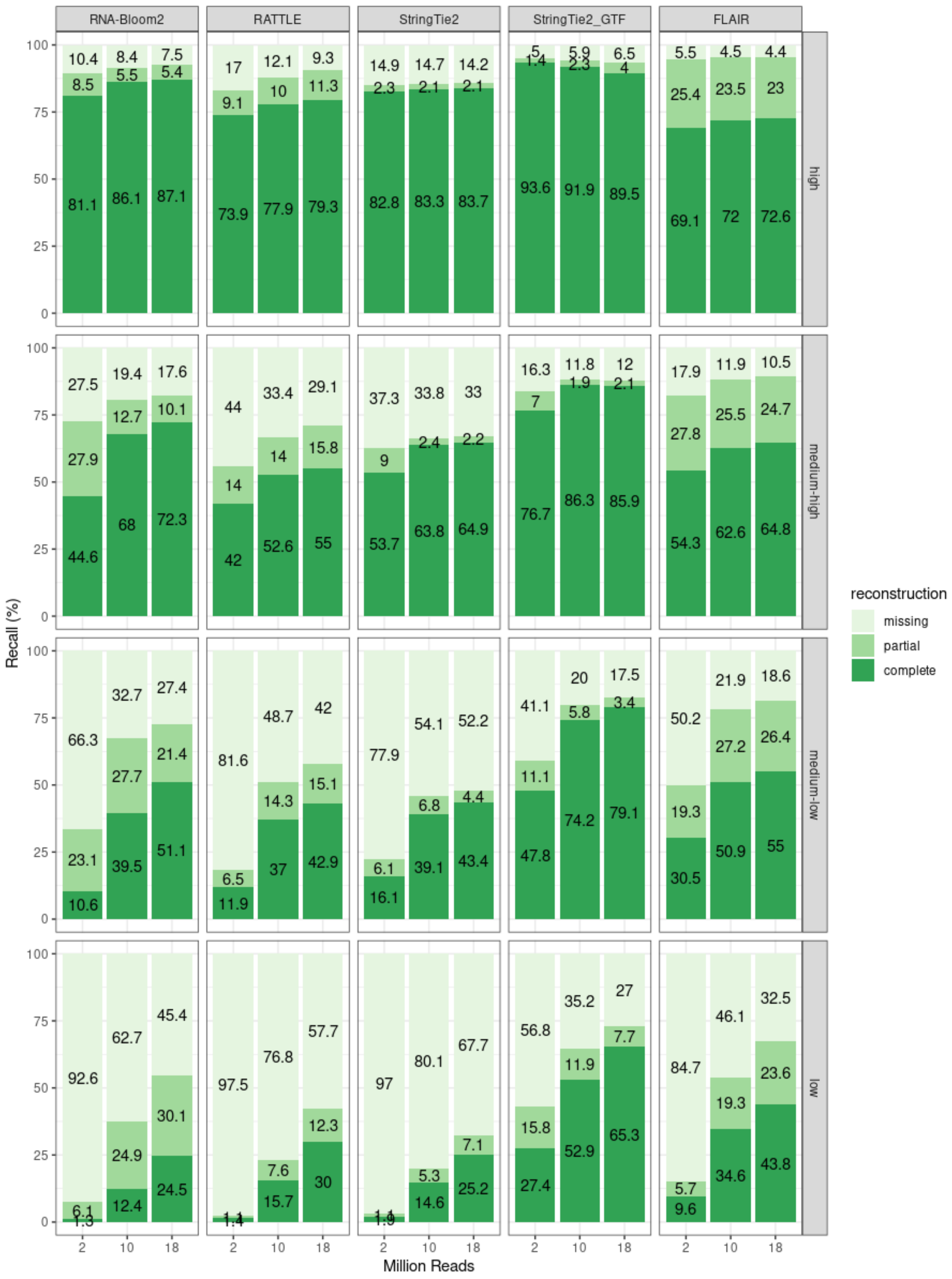

Supplementary Figure 3. Expression-stratified assembly recall on simulated dRNA data. Recall is categorized based on transcript reconstruction levels: missing, partial, and complete. Transcript expression levels are split into quartiles: low, medium-low, medium-high, and high. Source data are provided as a Source Data file.

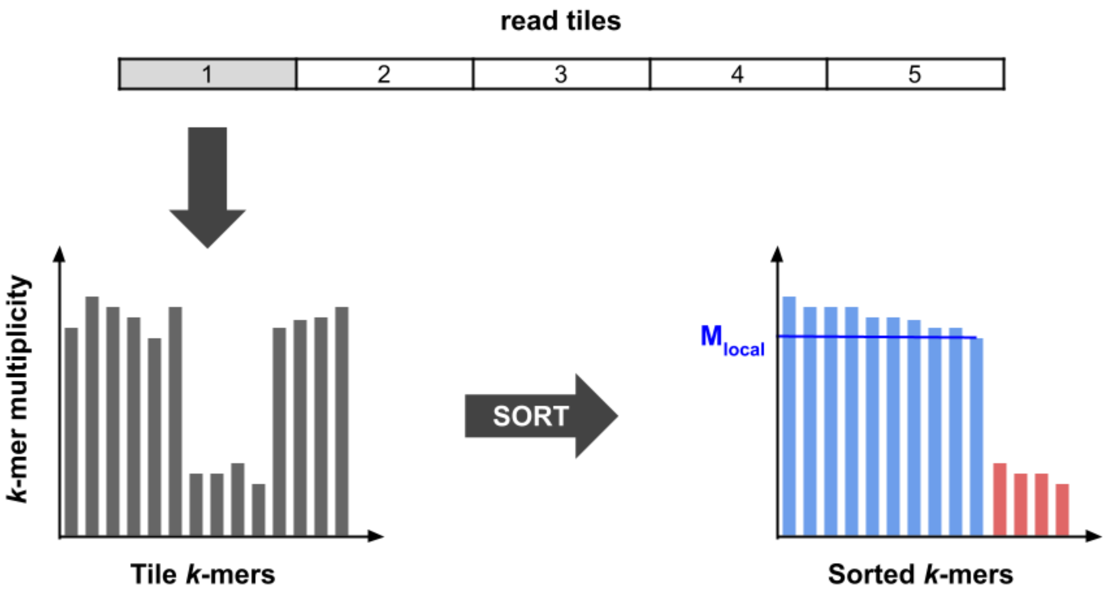

Supplementary Figure 4. *K*-mer multiplicity threshold for error correction.
In each tile of a read, the *k*-mer multiplicity threshold for distinguishing strong and weak *k*-mers is dynamically set to the maximum of the fixed global threshold and the local threshold. The global threshold is defaulted to 2, unless specified otherwise by the `-c` option in RNA-Bloom2. To calculate the local threshold (M_{local}) for a tile, all *k*-mer multiplicity values within the tile are sorted in descending order and two consecutive values are evaluated at a time to search for the local threshold. When the second value is less than half of the first value, the local threshold is set to the first value and the search terminates. If no such local threshold is found, then the *k*-mer multiplicity threshold is set to the global threshold.

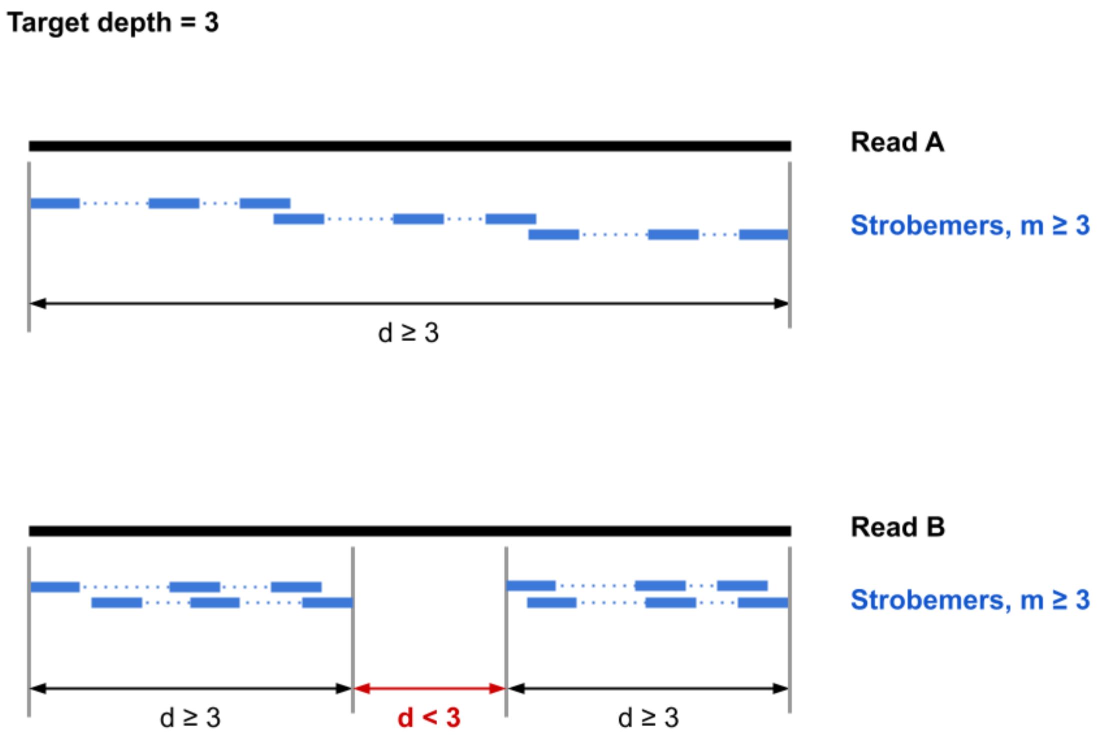

Supplementary Figure 5. Read depth tracking with strobemers.
Read A has an overlapping chain of strobemers with multiplicities at least 3, which implies that the other reads previously kept would already span across read A at least 3 times. Therefore, read A will not be kept during digital normalization. Since the middle region of read B is not spanned by any strobemers with multiplicity at least 3, the target depth of 3 has not been reached for this region. Therefore, read B will be kept during digital normalization.

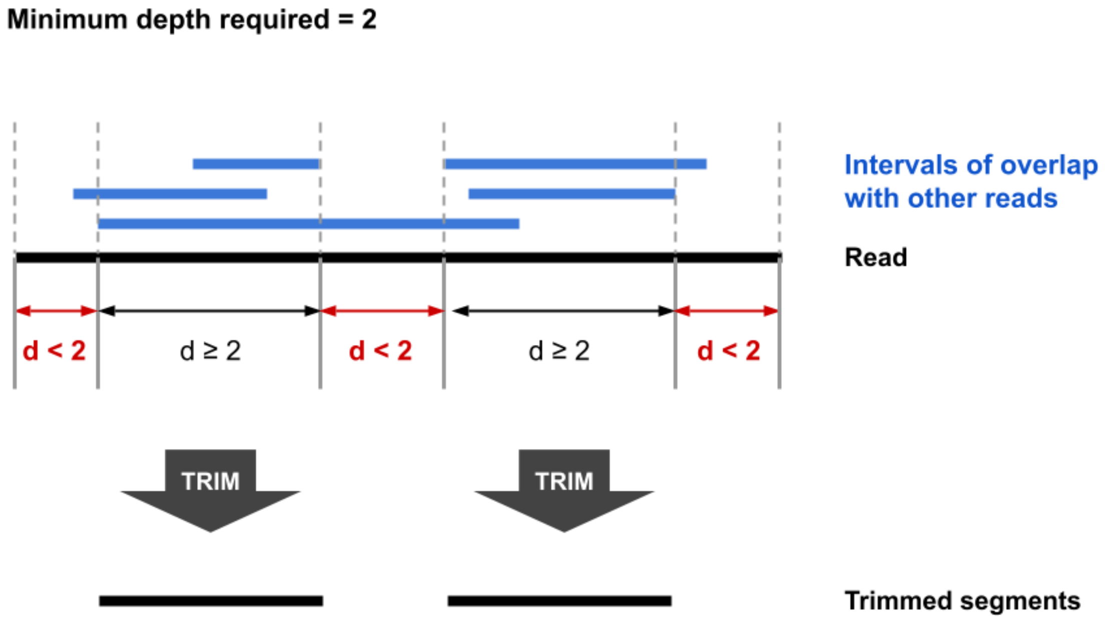

Supplementary Figure 6. Trimming and splitting based on read depth.
The read depth across a given read can be tallied using read-to-read overlaps. Low-depth regions on both ends are trimmed. A read may also be split at internal low-depth region(s) into shorter segments.

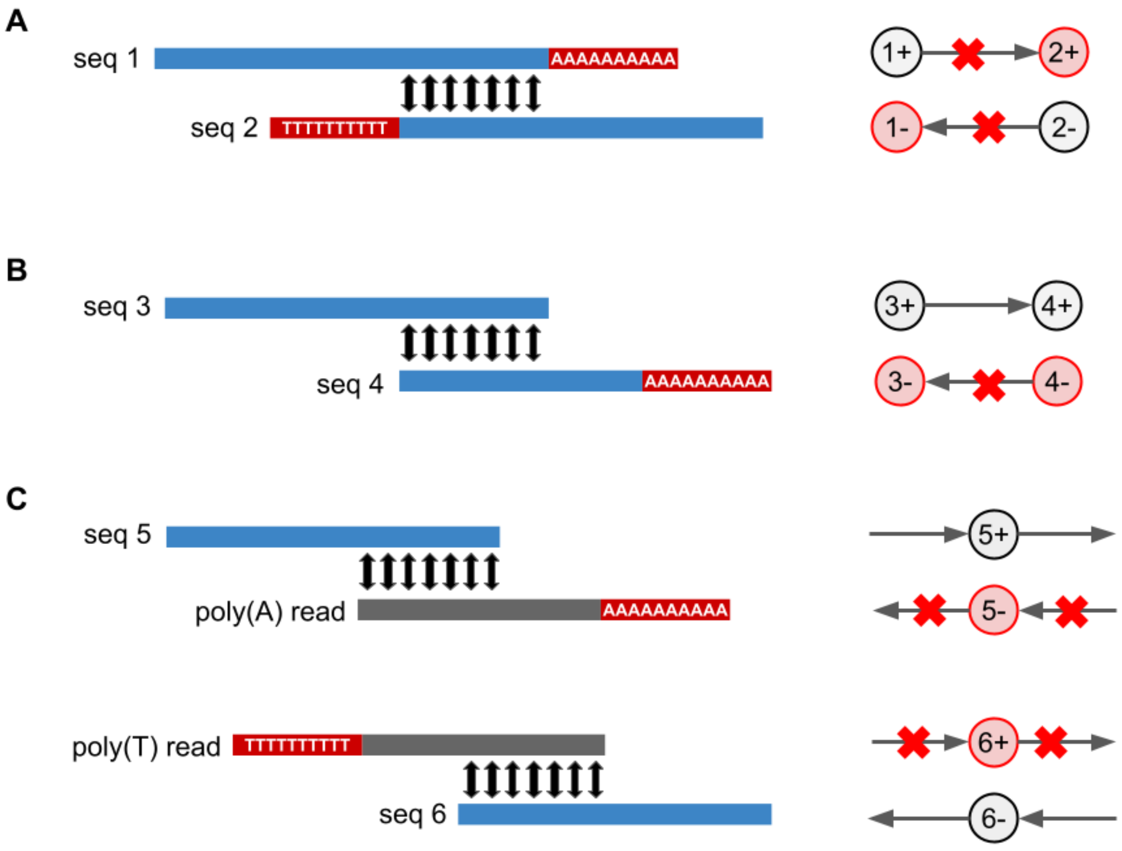

Supplementary Figure 7. Overlap graph pruning based on poly(A) information.
(A) A dovetail alignment with short overhangs (by default, max. 50-nt) are allowed and an edge is created in the overlap graph as a result. If the overhangs contain a poly(A) tail and a poly(T) head, then this is an indication of transcripts derived from overlapping antisense genes. Therefore, any vertices and edges for the opposite strand are pruned from the overlap graph.
(B) If a sequence is solely overlapping with another sequence containing a poly(A) tail, then the vertices and edges for the opposite strand are removed.
(C) If a sequence is only aligned by poly(A) reads, then this sequence is already in the correct orientation. If a sequence is only aligned by poly(T) reads, then this sequence should be reverse-complemented.

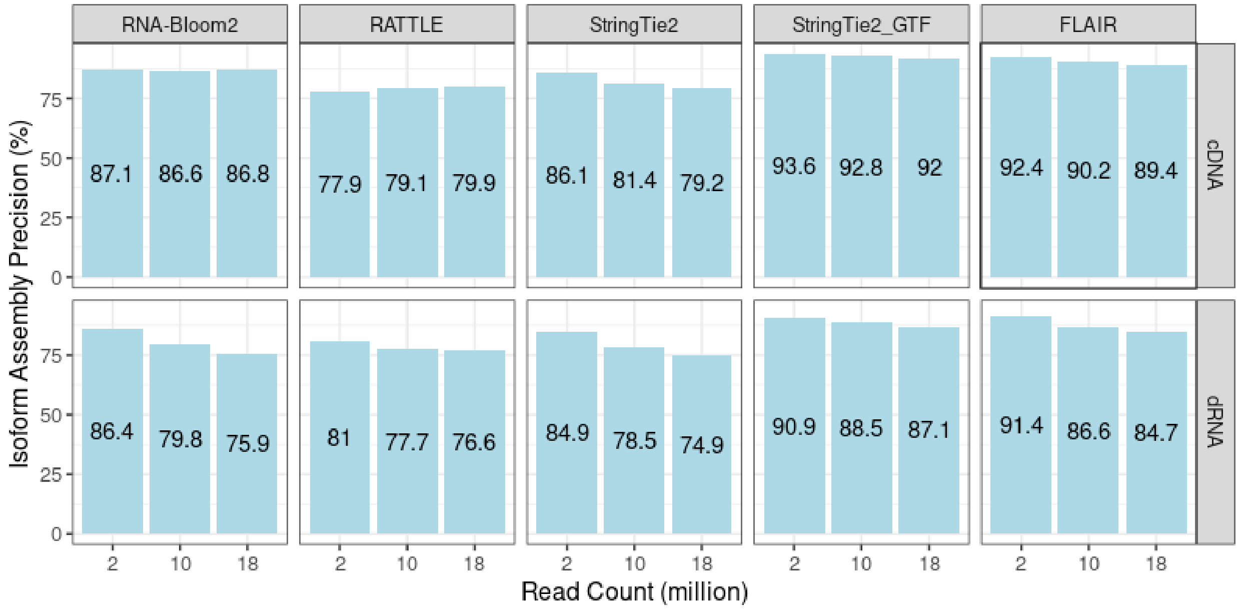

Supplementary Figure 8. Isoform assembly precision on simulated data.
The isoform assembly precision is calculated based on the isoform classification from SQANTI3. The number indicated in each bar represents the isoform assembly precision (%). Source data are provided as a Source Data file.

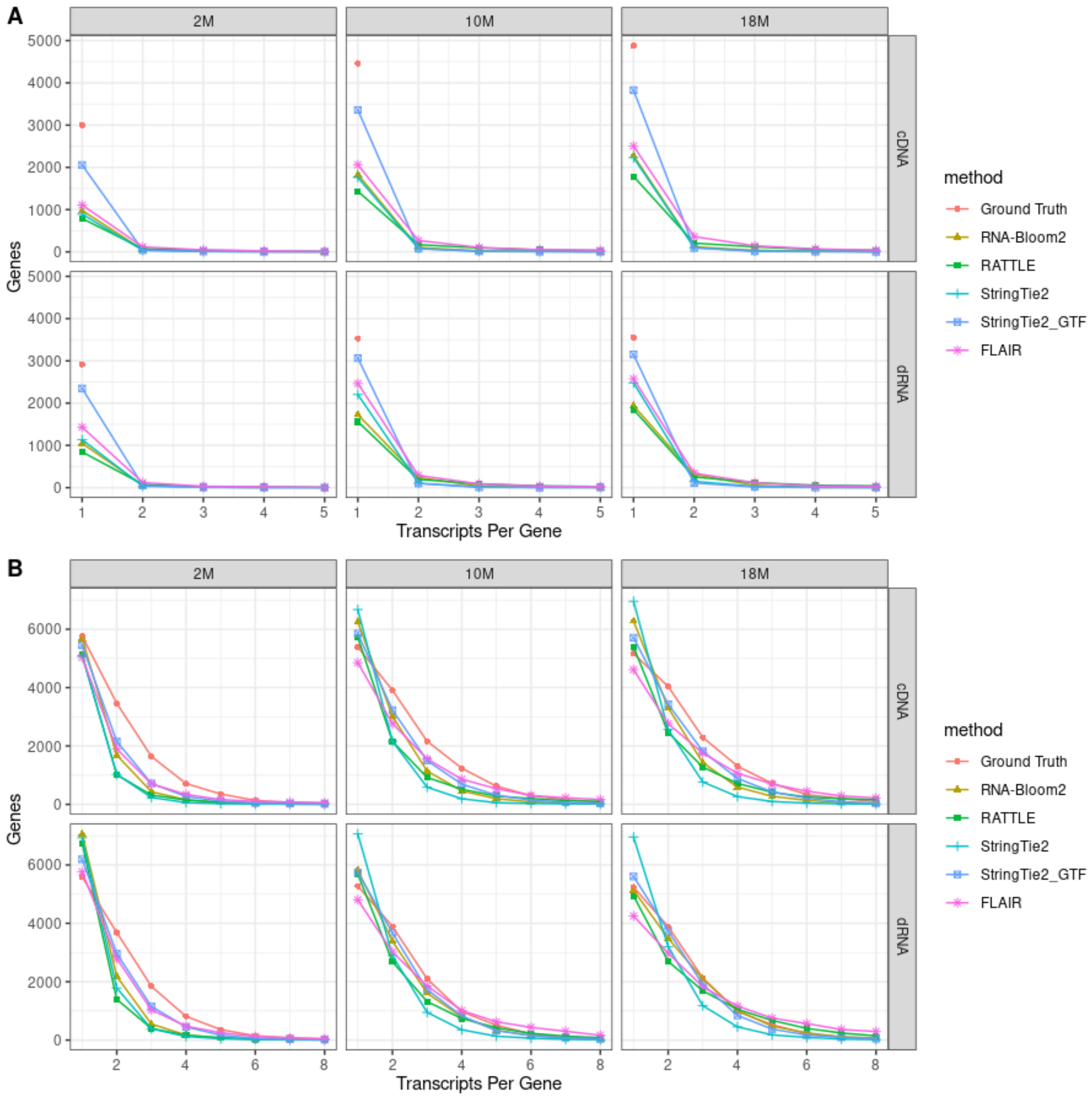

Supplementary Figure 9. Transcripts detected per gene in simulated data.
(A) Transcripts per gene for single-transcript genes (B) Transcripts per gene for multi-transcript genes. For assembly methods, the numbers of contigs with complete or partial reconstruction of true positive transcripts are presented. Source data are provided as a Source Data file.

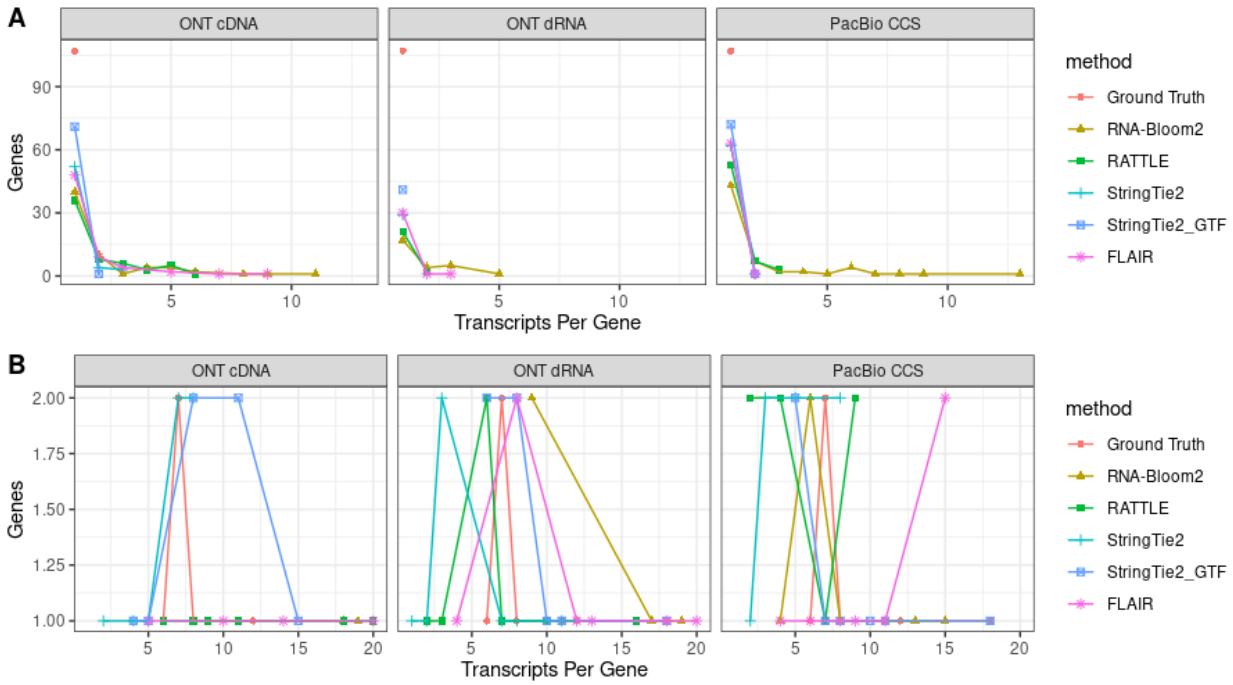

Supplementary Figure 10. Transcripts detected per gene in spike-in control data. (A) Transcripts per gene for single-transcript genes (B) Transcripts per gene for multi-transcript genes. For assembly methods, the numbers of contigs with complete or partial reconstruction of true positive transcripts are presented. Source data are provided as a Source Data file.

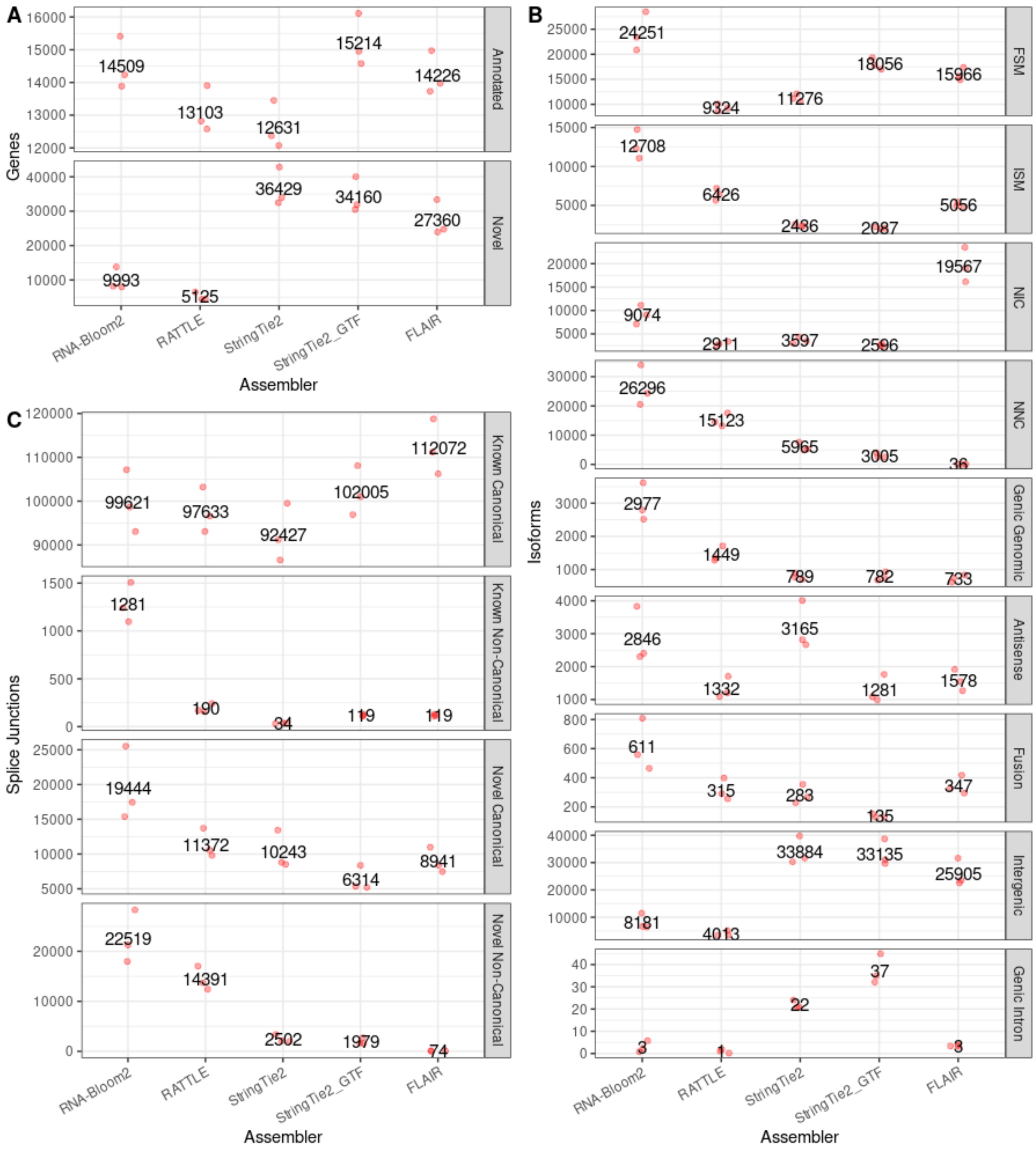

Supplementary Figure 11. SQANTI3 results for three ONT cDNA mouse replicates. (A) Gene categories. (B) Isoform categories. FSM, ISM, NIC, and NNC refer to full splice match, incomplete splice match, novel in-catalog, and novel not-in-catalog, respectively. (C) Splice junction categories. Data points for each replicate are colored in red; the text labels display the mean across replicates for each assembler. Source data are provided as a Source Data file.

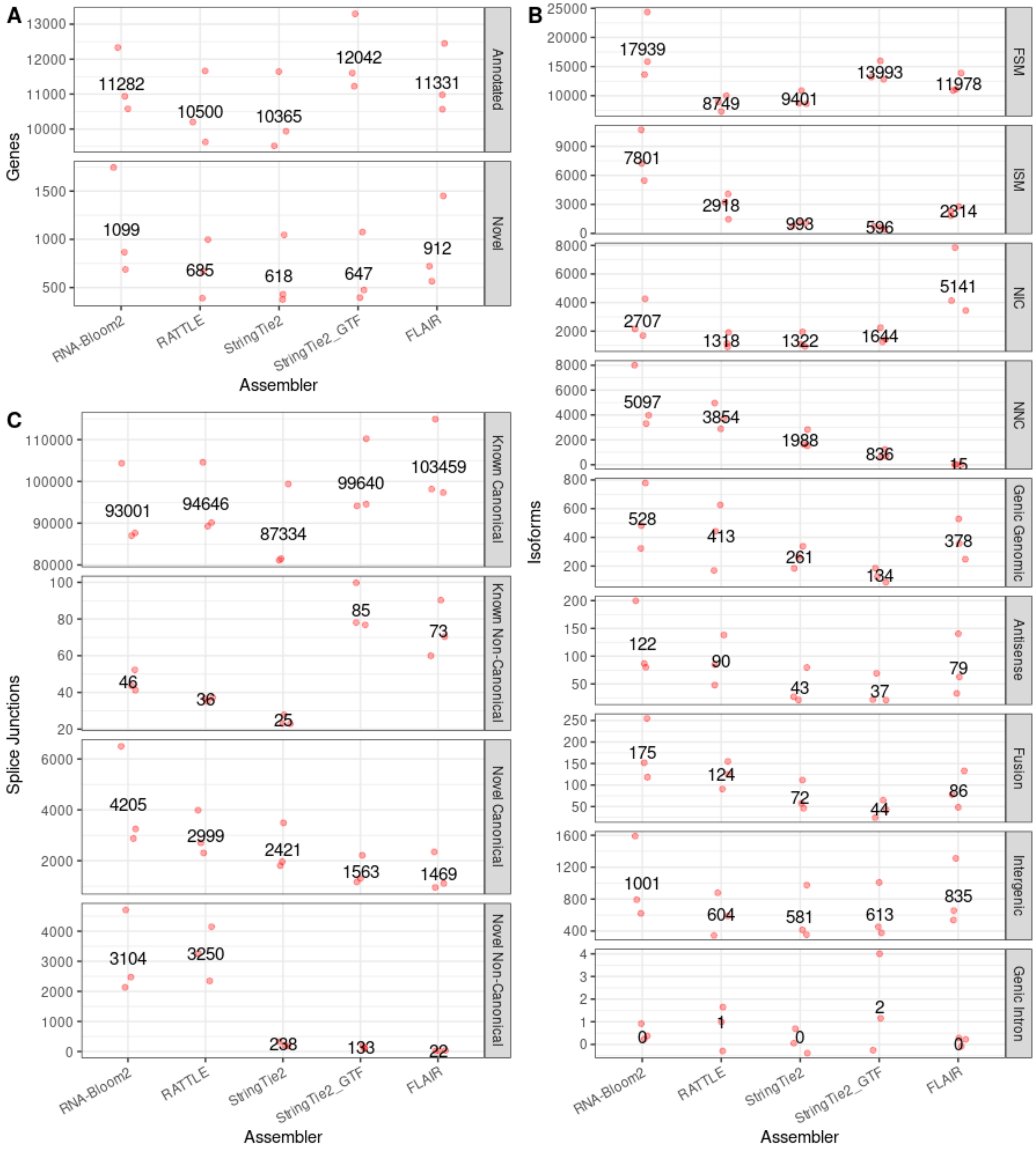

Supplementary Figure 12. SQANTI3 results for three ONT dRNA mouse replicates. (A) Gene categories. (B) Isoform categories. FSM, ISM, NIC, and NNC refer to full splice match, incomplete splice match, novel in-catalog, and novel not-in-catalog, respectively. (C) Splice junction categories. Data points for each replicate are colored in red; the text labels display the mean across replicates for each assembler. Source data are provided as a Source Data file.

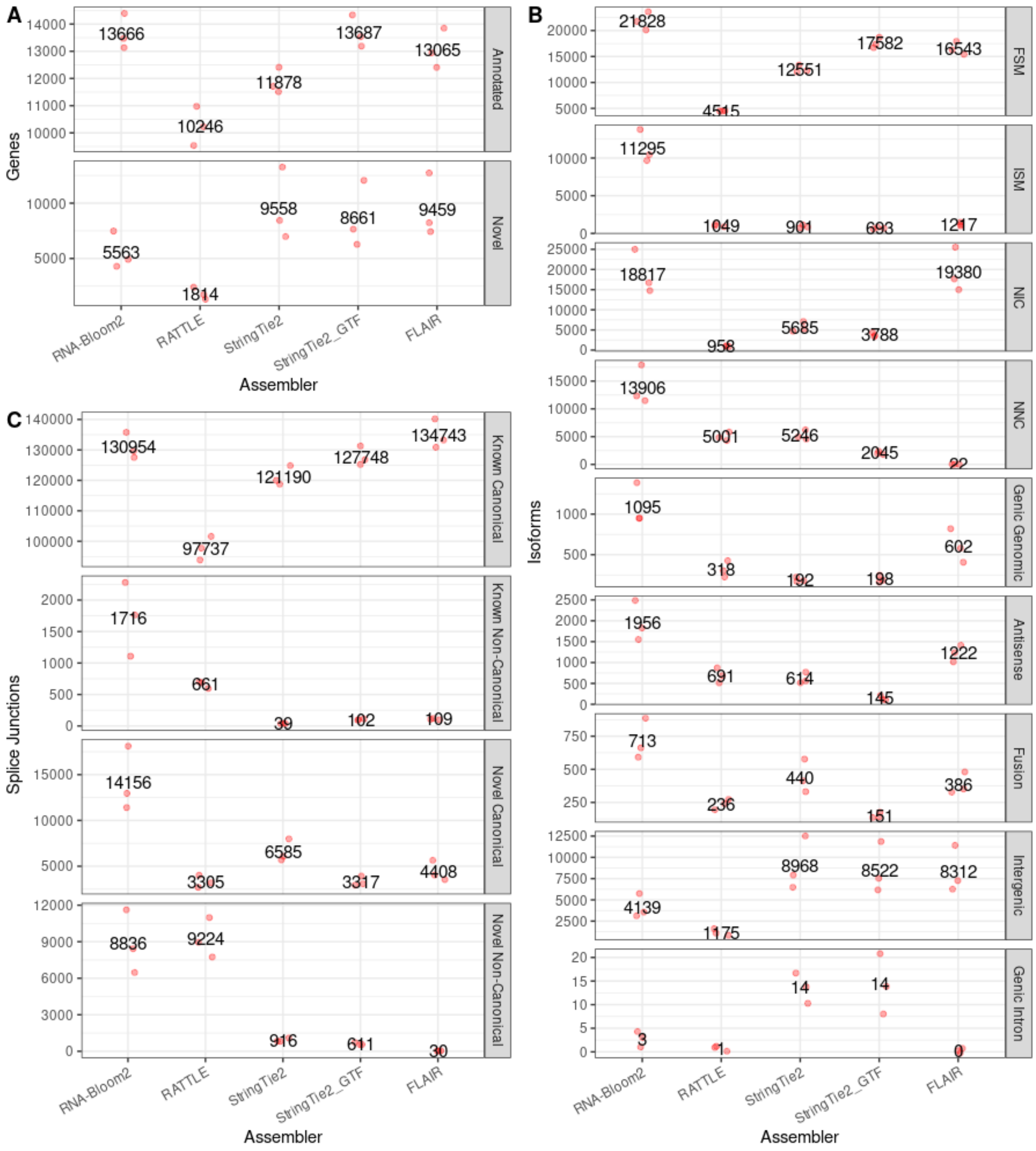

Supplementary Figure 13. SQANTI3 results for three PacBio CCS mouse replicates. (A) Gene categories. (B) Isoform categories. FSM, ISM, NIC, and NNC refer to full splice match, incomplete splice match, novel in-catalog, and novel not-in-catalog, respectively. (C) Splice junction categories. Data points for each replicate are colored in red; the text labels display the mean across replicates for each assembler. Source data are provided as a Source Data file.

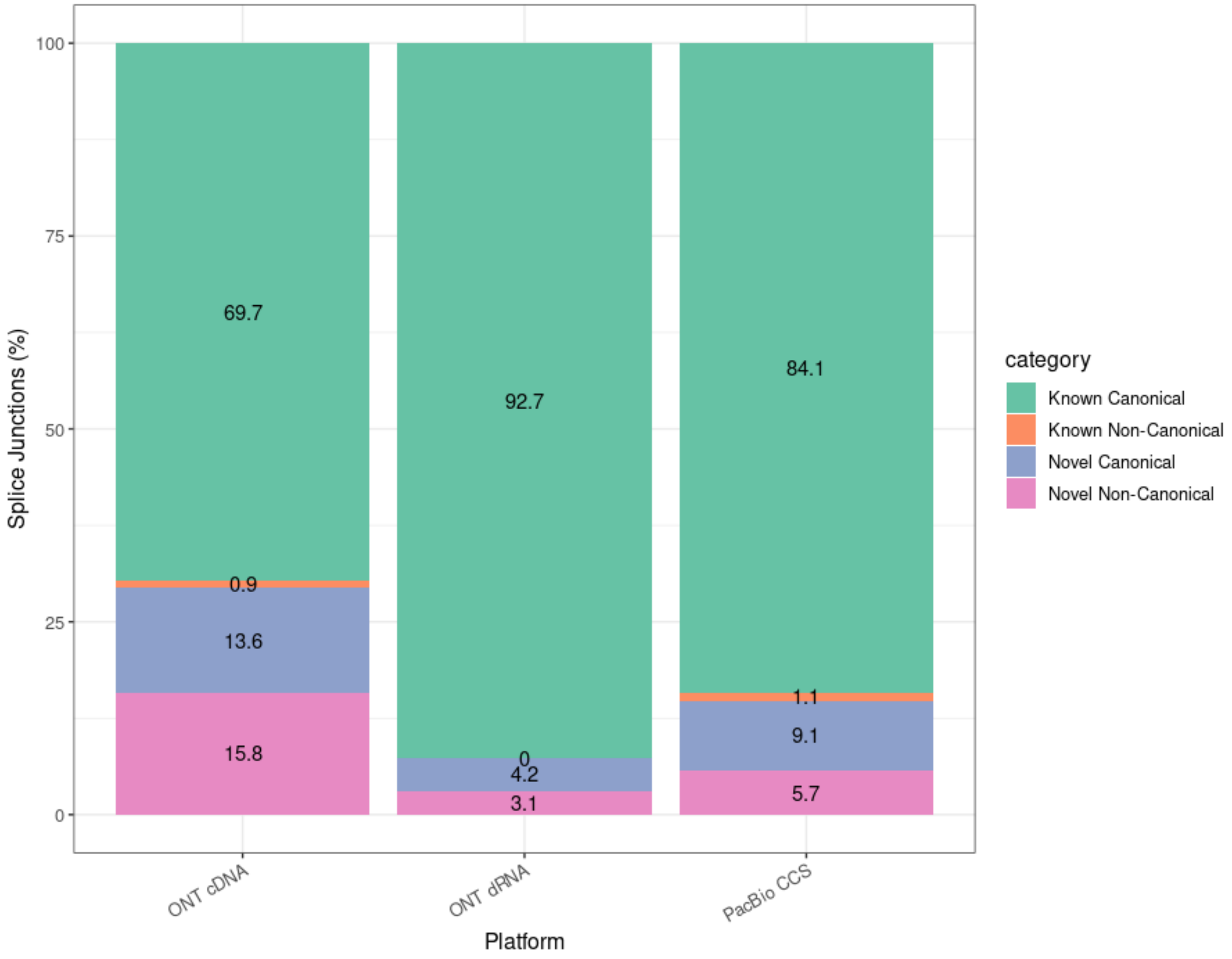

Supplementary Figure 14. Splice junction proportions in RNA-Bloom2 assemblies.
The mean proportion of splice junctions in RNA-Bloom2 assemblies in three mouse replicates across three sequencing platforms: ONT cDNA, ONT dRNA, and PacBio CCS. The classification of splice junctions is reported by SQANTI3. Source data are provided as a Source Data file.

Supplementary Methods

Supplementary Method 1. Adapter trimming for LRGASP data.

ONT cDNA reads (Pychopper v2.5.0):

```
python cdna_classifier.py -t 24 -m edlib -Y 20000 \
       -b primer_data/PCS110_primers.fas \
       -S pychopper_stats.tsv \
       -r pychopper_report.pdf \
       -u pychopper_unclassified.fastq \
       -w pychopper_rescued.fastq \
       -K pychopper_qcfail.fastq \
       ENCFF232YSU.fastq pychopper_fulllength.fastq
```

Illumina reads (Trimmomatic v0.39):

```
java -jar trimmomatic-0.39.jar PE -phred33 \
       IN_1.fastq IN_2.fastq \
       OUT_PE_1.fastq OUT_SE_1.fastq \
       OUT_PE_2.fastq OUT_SE_2.fastq \
       ILLUMINACLIP:adapters.fa:2:30:10 \
       LEADING:3 TRAILING:3 SLIDINGWINDOW:4:15 MINLEN:25
```

Supplementary Method 2. Measuring error rates.

Trans-NanoSim v3.1.0:

```
python read_analysis.py transcriptome -t 16 \
       --no_intron_retention –no_model_fit \
       -rg lrgasp_grcm39_sirvs.fasta.gz \
       -rt lrgasp_gencode_vM27_sirvs.fa \
       -i reads.fastq -o training
```

Error rates are reported in `training_error_rate.tsv`.

Supplementary Method 3. Measuring alignment rates.

Align read to genome (minimap2 2.24-r1122):

```
minimap2 -t 16 -a -x splice lrgasp_grcm39_sirvs.fasta.gz READS.fastq | \
       samtools sort -T ./tmp -O bam -o aln.bam
samtools index aln.bam
samtools flagstat aln.bam > aln.bam.flagstat
```

Align read to RNA-Bloom2 assembly (minimap2 2.24-r1122):

```
minimap2 -t 16 -a -x map-ont rnabloom.transcripts.fa READS.fastq | \
       samtools sort -T ./tmp -O bam -o aln.bam
samtools index aln.bam
samtools flagstat aln.bam > aln.bam.flagstat
```

Supplementary Method 4. Simulation of cDNA and dRNA reads.

For cDNA read simulation, adapter-trimmed reads (See Supplementary Method 1) are used in the subsequent steps. Since no adapters were found in dRNA reads, raw reads were used for dRNA read simulation.

<u>Expression quantification (Trans-NanoSim v3.1.0):</u>

```
python read_analysis.py quantify -t 16 -e trans \
       -rt lrgasp_gencode_vM27_sirvs.fa \
       -i pychopper_fulllength.fastq -o tns
```

<u>Characterization and simulation (Seqtk 1.3-r106, Trans-NanoSim v3.1.0):</u>

```
seqtk sample pychopper_fulllength.fastq 1000000 > sample.fastq

python read_analysis.py transcriptome -t 16 --no_intron_retention \
       -rg lrgasp_grcm39_sirvs.fasta.gz \
       -rt lrgasp_gencode_vM27_sirvs.fa \
       -i sample.fastq -o training

python simulator.py transcriptome -t 16 --no_model_ir --fastq \
       -b guppy -r cDNA_1D2 \
       -rg lrgasp_grcm39_sirvs.fasta.gz \
       -rt lrgasp_gencode_vM27_sirvs.fa \
       -e tns_transcriptome_quantification.tsv \
       --polya polya_transcript_ids.txt \
       -c training -o sim25M -n 25000000
```

(use `-r dRNA` instead of `-r cDNA_1D2` for simulating direct RNA reads)

<u>Subsample to 2, 10, 18 million reads (Seqtk 1.3-r106):</u>

```
seqtk sample sim25M_aligned_reads.fastq 2000000 > sample.2M.fastq
seqtk sample sim25M_aligned_reads.fastq 10000000 > sample.10M.fastq
seqtk sample sim25M_aligned_reads.fastq 18000000 > sample.18M.fastq
```

Supplementary Method 5. Extraction of spike-in control reads.

All three replicates of the mouse ES sample from LRGASP were used for each platform:

	ONT cDNA	ONT dRNA	PacBio CCS
**File accessions**	ENCFF232YSU<br>ENCFF288PBL<br>ENCFF683TBO	ENCFF349BIN<br>ENCFF412NKJ<br>ENCFF765AEC	ENCFF313VYZ<br>ENCFF667VXS<br>ENCFF874VSI

For ONT cDNA data, adapter-trimmed reads ("fulllength" and "rescued" FASTQs from Pychopper) are used (See Supplementary Method 1). For ONT dRNA and PacBio CCS data, raw reads are used.

Align reads to LRGASP mouse reference genome (minimap2 2.24-r1122, samtools 1.14):

```
minimap2 -x splice -a --MD -L -Y -t 47 \
	lrgasp_grcm39_sirvs.fasta.gz reads.fastq | \
	samtools sort -T ./tmp -O bam -o aln.bam

samtools index aln.bam
```

Extract uniquely aligned reads to spike-in sequences (samtools 1.14):

```
regions="SIRV1 SIRV2 SIRV3 SIRV4 SIRV5 SIRV6 SIRV7 SIRV4001 SIRV4002 SIRV4003 SIRV6001
SIRV6002 SIRV6003 SIRV8001 SIRV8002 SIRV8003 SIRV10001 SIRV10002 SIRV10003 SIRV12001
SIRV12002 SIRV12003 ERCC-00002 ERCC-00003 ERCC-00004 ERCC-00009 ERCC-00012 ERCC-00013
ERCC-00014 ERCC-00016 ERCC-00017 ERCC-00019 ERCC-00022 ERCC-00024 ERCC-00025
ERCC-00028 ERCC-00031 ERCC-00033 ERCC-00034 ERCC-00035 ERCC-00039 ERCC-00040
ERCC-00041 ERCC-00042 ERCC-00043 ERCC-00044 ERCC-00046 ERCC-00048 ERCC-00051
ERCC-00053 ERCC-00054 ERCC-00057 ERCC-00058 ERCC-00059 ERCC-00060 ERCC-00061
ERCC-00062 ERCC-00067 ERCC-00069 ERCC-00071 ERCC-00073 ERCC-00074 ERCC-00075
ERCC-00076 ERCC-00077 ERCC-00078 ERCC-00079 ERCC-00081 ERCC-00083 ERCC-00084
ERCC-00085 ERCC-00086 ERCC-00092 ERCC-00095 ERCC-00096 ERCC-00097 ERCC-00098
ERCC-00099 ERCC-00104 ERCC-00108 ERCC-00109 ERCC-00111 ERCC-00112 ERCC-00113
ERCC-00116 ERCC-00117 ERCC-00120 ERCC-00123 ERCC-00126 ERCC-00130 ERCC-00131
ERCC-00134 ERCC-00136 ERCC-00137 ERCC-00138 ERCC-00142 ERCC-00143 ERCC-00144
ERCC-00145 ERCC-00147 ERCC-00148 ERCC-00150 ERCC-00154 ERCC-00156 ERCC-00157
ERCC-00158 ERCC-00160 ERCC-00162 ERCC-00163 ERCC-00164 ERCC-00165 ERCC-00168
ERCC-00170 ERCC-00171"

samtools view -h -F 0x800 aln.bam ${regions} | \
	grep -v 'SA:Z:' | \
	samtools view -hSu | \
	samtools sort -n -O BAM | \
	samtools fastq -n -c 6 -0 sirv_ercc.fastq -
```

Supplementary Method 6. Assembly of simulated data, spike-in data, and replicated data.

Machine specification for initial runs:
Intel(R) Xeon(R) CPU E5-2650 v4 @ 2.20GHz, 48 CPUs, 377 GB RAM

Machine specification for re-running RATTLE and FLAIR:
Intel(R) Xeon(R) CPU E7-8867 v3 @ 2.50GHz, 128 CPUs, 2.5 TB RAM

All software programs are run with 48 threads whenever possible.

<u>RNA-Bloom v2.0.0: (with ntCard 1.2.1, minimap2 2.24-r1122, Racon v1.4.20)</u>

For ONT cDNA data:

```
java -Xmx150g -jar RNA-Bloom.jar -t 48 -outdir OUTDIR \
       -long READS.fastq -fpr 0.005 -overlap 200 -length 150 \
       -lrop 0.7 -p 0.7 -lrrd 3
```

For ONT dRNA data:

```
java -Xmx150g -jar RNA-Bloom.jar -t 48 -outdir OUTDIR \
       -long READS.fastq -fpr 0.005 -overlap 200 -length 150 \
       -lrop 0.7 -p 0.7 -lrrd 3 -stranded
```

For PacBio CCS data:

```
java -Xmx150g -jar RNA-Bloom.jar -t 48 -outdir OUTDIR \
       -long READS.fastq -fpr 0.005 -overlap 200 -length 150 \
       -lrpb -lrrd 3
```

<u>RATTLE (commit d0f067947ca666b0ee9c360429a85a1bf6f5b09e; cloned from GitHub repository on April 13th, 2022):</u>

For ONT cDNA and PacBio CCS data:

```
rattle cluster -i reads.fastq -t 48 -o OUTDIR --iso

rattle cluster_summary -i READS.fastq -c OUTDIR/clusters.out \
       > OUTDIR/cluster_summary.tsv

mkdir OUTDIR/clusters
rattle extract_clusters -i READS.fastq -c OUTDIR/clusters.out \
       -o OUTDIR/clusters --fastq

rattle correct -i READS.fastq -c OUTDIR/clusters.out -o OUTDIR \
       -t 48 -r 3

rattle polish -i OUTDIR/consensi.fq -o OUTDIR -t 48
```

For ONT direct RNA data, the option `--rna` is included for the `cluster` and `polish` modules.
For replicated PacBio data, the option `--iso` is removed for the `cluster` module.

StringTie2 v2.2.1: (with minimap2 2.24-r1122, samtools v1.14, gffread v0.12.7)

```
minimap2 -t 48 -a -x splice REF.fasta READS.fastq | \
       samtools sort -T ./tmp -O bam -o aln.bam
```

```
samtools index aln.bam
```

```
stringtie -p 48 -L -c 3 -s 3 -m 150 -o assembly.gtf aln.bam
```

(For runs including the transcriptome annotation, add `-G ANNOTATION.gtf` to the above command)

```
gffread -w assembly.fasta -g REF.fasta assembly.gtf
```

FLAIR (commit e248d2cec3a0dc8c0c291da6f19b2abdd211d80e; cloned from GitHub repository on April 13th, 2022): (with minimap2 2.24-r1122, samtools v1.14)

```
python flair.py align -t 48 -g REF.fasta -r READS.fastq -v1.3
```

```
python flair.py correct -t 48 -g REF.fasta -q flair.aligned.bed --gtf ANNOTATION.gtf
```

```
python flair.py collapse -t 48 -g REF.fasta -r READS.fastq \
       -q flair_all_corrected.bed --temp_dir TMPDIR -s 3 --gtf ANNOTATION.gtf
```

For ONT direct RNA data, the option `--nvrna` is included for the `align` and `correct` modules.

Supplementary Method 7. Sitka spruce transcriptome analysis.

<u>Basecalling with Guppy v5.0.15:</u>

```
guppy_basecaller --input_path FAST5_DIR \
       --save_path OUTDIR \
       --recursive \
       -c dna_r9.4.1_450bps_hac_prom.cfg \
       --device cuda:0 cuda:1 cuda:2 cuda:3 \
       --compress_fastq
```

<u>Adapter-trimming with Porechop v0.2.4:</u>

Custom adapters added to Porechop's `adapters.py`:

```
           Adapter('oligo-dTVN',
                    start_sequence=('oligo-dTVN', 'TATCAACGCAGAGTACTTTT'),
                    end_sequence=('oligo-dTVN_rev', 'AAAAGTACTCTGCGTTGATA')),
           Adapter('TSO',
                    start_sequence=('TSO', 'TATCAACGCAGAGTACGGG'),
                    end_sequence=('TSO_rev', 'CCCGTACTCTGCGTTGATA')),
```

```
python porechop-runner.py \
       -i pass.fastq.gz \
       -o porechop.fastq \
       --check_reads 20000 --end_threshold 80 --end_size 100 \
       --min_trim_size 8 --extra_end_trim 0 --min_split_read_size 150 \
       --extra_middle_trim_good_side 0 --extra_middle_trim_bad_side 40
```

<u>Assembly with RNA-Bloom v2.0.0:</u>
(with ntCard 1.2.1, minimap2 2.24-r1122, Racon v1.4.20)

Long-read assembly:

```
java -Xmx150g -jar RNA-Bloom.jar -t 48 -outdir OUTDIR \
       -long PORECHOP.fastq -fpr 0.005 -overlap 200 -length 200 \
       -lrrd 2
```

Long-read assembly with hybrid error correction:

```
java -Xmx150g -jar RNA-Bloom.jar -t 48 -outdir OUTDIR \
       -long PORECHOP.fastq -fpr 0.005 -overlap 200 -length 200 \
       -left SHORT_READS_1.fastq -right SHORT_READS_2.fastq \
       -lrrd 2
```

<u>Assembly with RATTLE (commit d0f067947ca666b0ee9c360429a85a1bf6f5b09; cloned from GitHub repository on April 13th, 2022):</u>

```
rattle cluster -i PORECHOP.fastq -t 48 -o OUTDIR --iso

rattle cluster_summary -i PORECHOP.fastq -c OUTDIR/clusters.out \
       > OUTDIR/cluster_summary.tsv
```

```
mkdir OUTDIR/clusters
rattle extract_clusters -i PORECHOP.fastq -c OUTDIR/clusters.out \
       -o OUTDIR/clusters --fastq

rattle correct -i PORECHOP.fastq -c OUTDIR/clusters.out -o OUTDIR \
       -t 48 -r 2

rattle polish -i OUTDIR/consensi.fq -o OUTDIR -t 48
```

BUSCO benchmarking with BUSCO v5.3.2:

```
busco -i SEQUENCES.fasta \
       -o OUTDIR \
       -l embryophyta_odb10 \
       -m transcriptome \
       -c 12
```

Alignment of paired-end reads with STAR v2.7.10a:

```
STAR --runThreadN 48 \
       --genomeDir STAR_INDEX \
       --readFilesIn READS_R1.fastq.gz,... READS_R2.fastq.gz,... \
       --outFileNamePrefix OUTPREFIX \
       --alignSJoverhangMin 8 \
       --alignSJDBoverhangMin 1 \
       --outFilterType BySJout \
       --outSAMunmapped Within \
       --outFilterMultimapNmax 20 \
       --outFilterMismatchNoverLmax 0.04 \
       --outFilterMismatchNmax 999 \
       --alignIntronMin 20 --alignIntronMax 1000000 \
       --alignMatesGapMax 1000000 \
       --sjdbScore 1 \
       --genomeLoad NoSharedMemory \
       --outSAMtype BAM SortedByCoordinate \
       --twopassMode Basic \
       --readFilesCommand zcat \
       --chimOutType WithinBAM --chimSegmentMin 40
```

Note that this task used ~1TB of memory!

Gene structure annotation with PASA v2.5.2:
(with minimap2 2.24-r1122, samtools v1.15.1)

```
# extract assembly sequence IDs
grep '^>' rnabloom.transcripts.fa | sed -e 's/^>//g' -e 's/ .*//g' > FL_accs.txt

PASApipeline-v2.5.2/bin/seqclean rnabloom.transcripts.fa

PASApipeline-v2.5.2/Launch_PASA_pipeline.pl -c alignAssembly.config \
       --CPU 24 -C -R -g combined_genome_assembly.fasta \
       --ALT_SPLICE -t rnabloom.transcripts.fa.clean \
       -T -u rnabloom.transcripts.fa \
       -f FL_accs.txt \
       --ALIGNERS minimap2 --MAX_INTRON_LENGTH 1000000 \
       --TRANSDECODER

PASApipeline-v2.5.2/scripts/pasa_asmbls_to_training_set.dbi \
```

```
        --pasa_transcripts_fasta database.sqlite.assemblies.fasta \
        --pasa_transcripts_gff3 database.sqlite.pasa_assemblies.gff3
```

Functional annotation with EnTAP v0.10.8-beta:

```
EnTAP --runP \
        -i rnabloom.transcripts.fa \
        -d uniprot-2021_03_swissprot-plant-proteins.dmnd \
        -d uniref90_reformatted.dmnd \
        -d odb10-plant-proteins_reformatted.dmnd \
        -d refseq_plant_hq.dmnd \
        -t 48 \
        --ini entap_config.ini
```

Similarity search for TPS, CYP, and NAC peptides with BLAST 2.2.31+:

```
blastp -db entap_outfiles.Transdecoder.processed.complete_genes \
      -query KNOWN_SPRUCE_TPS_CYP_NAC_PEPTIDES.fasta \
      -out Blast_hits.out \
      -outfmt "6 qseqid sseqid pident length mismatch gapopen qstart qend
sstart send evalue bitscore qcovs qcovhsp"
```

Supplementary Method 8. Edge filtering in the overlap graph.

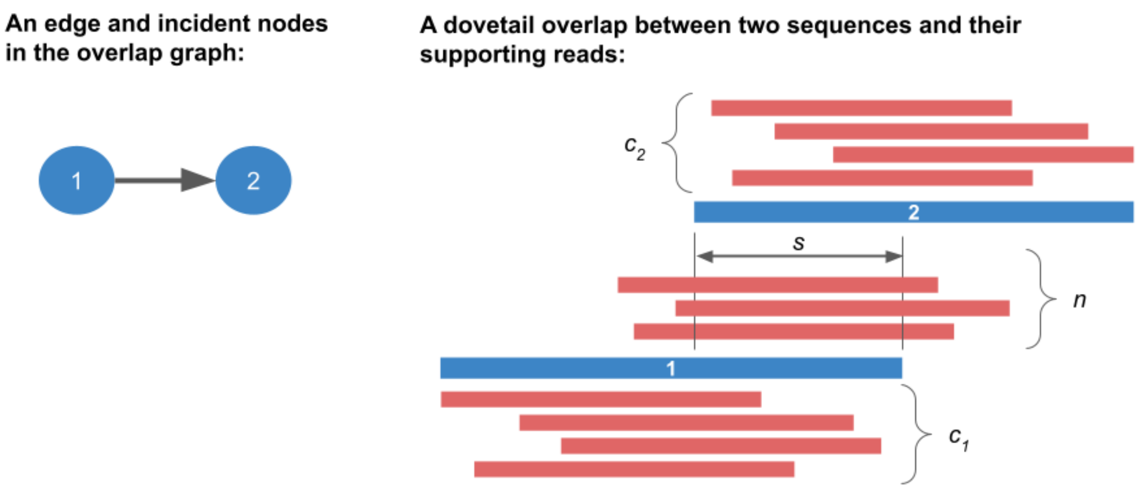

Each edge in the overlap graph represents the dovetail overlap between two sequences (i.e. the two incident nodes of the edge). An edge can be evaluated based on:

R = the length distribution for all corrected reads
s = the size of the overlap between sequences 1 and 2
n = the number of reads spanning across the overlap entirely
c_1 = the length-normalized read count of sequence 1
c_2 = the length-normalized read count of sequence 2

R is extracted during the error correction stage. s is defined for each edge when the overlap graph is constructed according to the overlaps between reads. n, c_1, and c_2 are determined by parsing the read alignments from the polishing stage. The length-normalized read count for a sequence is calculated as the total number of aligned bases in the aligned reads divided by the length of the sequence.

If $n \geq \min(c_1, c_2)$, then the edge is kept.
Otherwise, a one-tailed binomial test is applied to evaluate the statistical significance of observing this edge. R is used to calculate, q, the probability of observing a read shorter than s. From the binomial distribution, $B(\min(c_1, c_2), 1 - q)$, the p-value for n is calculated. The edge is removed if p-value < 0.001.

Supplementary Method 9. Determining gene representation in sequencing reads.

For a given set of reads, transcript expression is quantified with Trans-NanoSim v3.1.0:

```
python read_analysis.py quantify \
       -e trans -t 16 -o exp \
       -i reads.fastq.gz \
       -rt lrgasp_gencode_vM27_sirvs.fa
```

The expression of each gene is calculated as the sum of its transcript isoform expression levels (i.e. TPM). A gene is designated as represented by a given set of reads if its expression level is above zero (i.e. TPM > 0).

Supplementary Method 10. Running RNA-Bloom2 and isONcorrect.

Since isONcorrect does not filter reads based on read depth, the read-depth threshold (i.e. `-lrrd`) for assembly in RNA-Bloom2 has been reduced to 1 (from 3).

RNA-Bloom v2.0.0: (with ntCard 1.2.1, minimap2 2.24-r1122, Racon v1.4.20)

For ONT cDNA data:

```
java -Xmx150g -jar RNA-Bloom.jar -t 48 -outdir OUTDIR \
       -long READS.fastq -fpr 0.005 -overlap 200 -length 150 \
       -lrop 0.7 -p 0.7 -lrrd 1
```

For ONT dRNA data:

```
java -Xmx150g -jar RNA-Bloom.jar -t 48 -outdir OUTDIR \
       -long READS.fastq -fpr 0.005 -overlap 200 -length 150 \
       -lrop 0.7 -p 0.7 -lrrd 1 -stranded
```

isONcorrect v0.0.8: (with isONclust v0.0.6.1)

```
isONclust --t 48 --ont --fastq READS.fastq --outfolder ./clustering --consensus

isONclust write_fastq --N 1 --clusters ./clustering/final_clusters.tsv \
    --fastq READS.fastq --outfolder ./clustering/fastq_files

run_isoncorrect --t 48 --fastq_folder ./clustering/fastq_files \
    --outfolder ./correction/

touch OUTPUT.fastq
OUTFILES="./correction/"*"/corrected_reads.fastq"
for f in $OUTFILES
do
  echo $f
  cat $f >> OUTPUT.fastq
done
```

Supplementary Method 11. Running SQANTI3 v5.1

For RNA-Bloom2 and RATTLE:

```
tss=SQANTI3-5.1/data/ref_TSS_annotation/mouse.refTSS_v3.1.GRCm39.bed
polya_motifs=SQANTI3-5.1/data/polyA_motifs/mouse_and_human.polyA_motif.txt

python SQANTI3-5.1/sqanti3_qc.py ASSEMBLY.fasta REF_GTF lrgasp_grcm39_sirvs.fasta \
        --fasta \
        --force_id_ignore \
        --CAGE_peak ${tss} \
        --polyA_motif_list ${polya_motifs} \
        --dir ${outdir} \
        --cpus 48 \
        --skipORF
```

For StringTie2, StringTie2_GTF, and FLAIR:

```
tss=SQANTI3-5.1/data/ref_TSS_annotation/mouse.refTSS_v3.1.GRCm39.bed
polya_motifs=SQANTI3-5.1/data/polyA_motifs/mouse_and_human.polyA_motif.txt

python SQANTI3-5.1/sqanti3_qc.py ASSEMBLY.gtf REF_GTF lrgasp_grcm39_sirvs.fasta \
        --force_id_ignore \
        --CAGE_peak ${tss} \
        --polyA_motif_list ${polya_motifs} \
        --dir ${outdir} \
        --cpus 48 \
        --skipORF
```

For simulated data, options `--CAGE_peak` and `--polyA_motif_list` are removed and a filtered annotation GTF is used for each set of reads corresponding to the isoforms that are simulated. For runs on mouse data replicates, the complete reference annotation GTF is used.
