# Supplementary material for: Reference-free assembly of long-read transcriptome sequencing data with RNA-Bloom2
Source: Nat Commun. 2023 May 22;14:2940. doi: 10.1038/s41467-023-38553-y (PMC10202958; doi:10.1038/s41467-023-38553-y)
Supplement: Supplementary file 3 — Reporting Summary [file 41467_2023_38553_MOESM3_ESM.pdf]

nature portfolio

Corresponding author(s): Ka Ming Nip, Inanc Birol

Last updated by author(s): Apr 29, 2023

Reporting Summary

Nature Portfolio wishes to improve the reproducibility of the work that we publish. This form provides structure for consistency and transparency in reporting. For further information on Nature Portfolio policies, see our Editorial Policies and the Editorial Policy Checklist.

Statistics

For all statistical analyses, confirm that the following items are present in the figure legend, table legend, main text, or Methods section.

n/a	Confirmed	
☒	☐	The exact sample size (*n*) for each experimental group/condition, given as a discrete number and unit of measurement
☒	☐	A statement on whether measurements were taken from distinct samples or whether the same sample was measured repeatedly
☒	☐	The statistical test(s) used AND whether they are one- or two-sided<br>*Only common tests should be described solely by name; describe more complex techniques in the Methods section.*
☒	☐	A description of all covariates tested
☒	☐	A description of any assumptions or corrections, such as tests of normality and adjustment for multiple comparisons
☐	☒	A full description of the statistical parameters including central tendency (e.g. means) or other basic estimates (e.g. regression coefficient) AND variation (e.g. standard deviation) or associated estimates of uncertainty (e.g. confidence intervals)
☒	☐	For null hypothesis testing, the test statistic (e.g. *F*, *t*, *r*) with confidence intervals, effect sizes, degrees of freedom and *P* value noted<br>*Give P values as exact values whenever suitable.*
☒	☐	For Bayesian analysis, information on the choice of priors and Markov chain Monte Carlo settings
☒	☐	For hierarchical and complex designs, identification of the appropriate level for tests and full reporting of outcomes
☒	☐	Estimates of effect sizes (e.g. Cohen's *d*, Pearson's *r*), indicating how they were calculated

Our web collection on statistics for biologists contains articles on many of the points above.

Software and code

Policy information about availability of computer code

Data collection	External software tools used:<br>Pychopper v2.5.0, Trimmomatic v0.39, Trans-NanoSim v3.1.0, minimap2 2.24-r1122, Seqtk 1.3-r106, samtools 1.14, ntCard 1.2.1, Racon v1.4.20, RATTLE commit d0f067947ca666b0ee9c360429a85a1bf6f5b09e, StringTie2 v2.2.1, gffread v0.12.7, FLAIR commit e248d2cec3a0dc8c0c291da6f19b2abdd211d80e, Guppy v5.0.15, Porechop v0.2.4, BUSCO v5.3.2, STAR v2.7.10a, PASA v2.5.2, EnTAP v0.10.8-beta, BLAST 2.2.31+, isONcorrect v0.0.8, isONclust v0.0.6.1, SQANTI3 v5.1
Data analysis	The source code of RNA-Bloom2 v2.0.0 is available at:<br>https://github.com/bcgsc/RNA-Bloom (see release version v2.0.0)<br><br>Scripts for bioinformatics analysis is available at:<br>https://github.com/bcgsc/rnabloom2_manuscript (see release version v0.0.1)<br>https://github.com/bcgsc/rnaseq_utils (see release version v0.0.2)

For manuscripts utilizing custom algorithms or software that are central to the research but not yet described in published literature, software must be made available to editors and reviewers. We strongly encourage code deposition in a community repository (e.g. GitHub). See the Nature Portfolio guidelines for submitting code & software for further information.

Data

Policy information about availability of data

All manuscripts must include a data availability statement. This statement should provide the following information, where applicable:

- Accession codes, unique identifiers, or web links for publicly available datasets
- A description of any restrictions on data availability
- For clinical datasets or third party data, please ensure that the statement adheres to our policy

Our simulated data for assembly benchmarking is available on Dryad at:
https://doi.org/10.5061/dryad.cc2fqz68w

Our rebasecalled ONT sequencing data for the Sitka spruce cDNA sample is available on NCBI Sequence Read Archive (SRA) with accession SRR19510936 [https://www.ncbi.nlm.nih.gov/sra/?term=SRR19510936].

The following are external public data used in the paper.

LRGASP Consortium GRCm39 based mouse reference genome is available on Synapse with accession syn25683365 [https://www.synapse.org/#!Synapse:syn25683365].

LRGASP Consortium GENCODE VM27-based mouse transcriptome annotation set is available on Synapse with accession syn25683629 [https://www.synapse.org/#!Synapse:syn25683629].

LRGASP Consortium sequencing data are available in the ENCODE Project repository with the accessions:
ENCFF349BIN [https://www.encodeproject.org/files/ENCFF349BIN/],
ENCFF412NKJ [https://www.encodeproject.org/files/ENCFF412NKJ/],
ENCFF765AEC [https://www.encodeproject.org/files/ENCFF765AEC/],
ENCFF232YSU [https://www.encodeproject.org/files/ENCFF232YSU/],
ENCFF288PBL [https://www.encodeproject.org/files/ENCFF288PBL/],
ENCFF683TBO [https://www.encodeproject.org/files/ENCFF683TBO/],
ENCFF313VYZ [https://www.encodeproject.org/files/ENCFF313VYZ/],
ENCFF667VXS [https://www.encodeproject.org/files/ENCFF667VXS/],
ENCFF874VSI [https://www.encodeproject.org/files/ENCFF874VSI/],
ENCFF696TCH [https://www.encodeproject.org/files/ENCFF696TCH/],
ENCFF751FTE [https://www.encodeproject.org/files/ENCFF751FTE/]

Illumina sequencing data for Sitka spruce from a previous study is available on NCBI SRA with accessions:
SRR5949081 [https://www.ncbi.nlm.nih.gov/sra/?term=SRR5949081],
SRR5949082 [https://www.ncbi.nlm.nih.gov/sra/?term=SRR5949082],
SRR5949083 [https://www.ncbi.nlm.nih.gov/sra/?term=SRR5949083],
SRR5949084 [https://www.ncbi.nlm.nih.gov/sra/?term=SRR5949084],
SRR5949085 [https://www.ncbi.nlm.nih.gov/sra/?term=SRR5949085],
SRR5949086 [https://www.ncbi.nlm.nih.gov/sra/?term=SRR5949086],
SRR5949087 [https://www.ncbi.nlm.nih.gov/sra/?term=SRR5949087],
SRR5949088 [https://www.ncbi.nlm.nih.gov/sra/?term=SRR5949088],
SRR5949089 [https://www.ncbi.nlm.nih.gov/sra/?term=SRR5949089],
SRR5949090 [https://www.ncbi.nlm.nih.gov/sra/?term=SRR5949090],
SRR5949091 [https://www.ncbi.nlm.nih.gov/sra/?term=SRR5949091],
SRR5949092 [https://www.ncbi.nlm.nih.gov/sra/?term=SRR5949092]

Human research participants

Policy information about studies involving human research participants and Sex and Gender in Research.

Reporting on sex and gender	Not applicable.
Population characteristics	Not applicable.
Recruitment	Not applicable.
Ethics oversight	Not applicable.

Note that full information on the approval of the study protocol must also be provided in the manuscript.

Field-specific reporting

Please select the one below that is the best fit for your research. If you are not sure, read the appropriate sections before making your selection.

☒ Life sciences ☐ Behavioural & social sciences ☐ Ecological, evolutionary & environmental sciences

For a reference copy of the document with all sections, see nature.com/documents/nr-reporting-summary-flat.pdf

Life sciences study design

All studies must disclose on these points even when the disclosure is negative.

Sample size	This is not relevant because no sample collection is performed in this study.
Data exclusions	Sequencing reads were only excluded if they are deemed as poor quality by the adapter-trimming softwares.
Replication	Our own data have no replicates. Only external data used in this study have replicates.
Randomization	Randomization is not applicable and there are no covariates to control. Each analysis was performed on a single biological sample and no inferences are made about the population.
Blinding	Blinding is not applicable because our study does not involve any clinical experiments.

Reporting for specific materials, systems and methods

We require information from authors about some types of materials, experimental systems and methods used in many studies. Here, indicate whether each material, system or method listed is relevant to your study. If you are not sure if a list item applies to your research, read the appropriate section before selecting a response.

Materials & experimental systems

n/a	Involved in the study	
☒	☐	Antibodies
☒	☐	Eukaryotic cell lines
☒	☐	Palaeontology and archaeology
☒	☐	Animals and other organisms
☒	☐	Clinical data
☒	☐	Dual use research of concern

Methods

n/a	Involved in the study	
☒	☐	ChIP-seq
☒	☐	Flow cytometry
☒	☐	MRI-based neuroimaging
